# The Genetic Architecture of Meat Quality Traits in a Crossbred Commercial Pig Population

**DOI:** 10.3390/foods11193143

**Published:** 2022-10-09

**Authors:** Zhanwei Zhuang, Jie Wu, Cineng Xu, Donglin Ruan, Yibin Qiu, Shenping Zhou, Rongrong Ding, Jianping Quan, Ming Yang, Enqin Zheng, Zhenfang Wu, Jie Yang

**Affiliations:** 1 National Engineering Research Center for Breeding Swine Industry, College of Animal Science, South China Agricultural University, Guangzhou 510642, China; 2 Guangdong Zhongxin Breeding Technology Co., Ltd., Guangzhou 511466, China; 3 College of Animal Science and Technology, Zhongkai University of Agriculture and Engineering, Guangzhou 510225, China; 4 Guangdong Provincial Key Laboratory of Agro-Animal Genomics and Molecular Breeding, South China Agricultural University, Guangzhou 510642, China; 5 Yunfu Subcenter of Guangdong Laboratory for Lingnan Modern Agriculture, Yunfu 527400, China

**Keywords:** DLY pigs, meat quality, meat color, GWAS, SNP

## Abstract

Meat quality is of importance in consumer acceptance and purchasing tendency of pork. However, the genetic architecture of pork meat quality traits remains elusive. Herein, we conducted genome-wide association studies to detect single nucleotide polymorphisms (SNPs) and genes affecting meat pH and meat color (L*, lightness; a*, redness; b*, yellowness) in 1518 three-way crossbred pigs. All individuals were genotyped using the GeneSeek Porcine 50K BeadChip. In sum, 30 SNPs and 20 genes are found to be associated with eight meat quality traits. Notably, we detect one significant quantitative trait locus (QTL) on SSC15 with a 143 kb interval for meat pH (pH_12h), together with the most promising candidate *TNS1*. Interestingly, two newly identified SNPs located in the *TTLL4* gene demonstrate the highest phenotypic variance of pH_12h in this QTL, at 2.67%. The identified SNPs are useful for the genetic improvement of meat quality traits in pigs by assigning higher weights to associated SNPs in genomic selection.

## 1. Introduction

Pork contributes to a large share of the world’s meat provision, especially in the Chinese market. People pay more attention to meat quality with the improvement in living standards. Pork quality can increase both customer purchasing tendency and pork consumption, and then enable the pig industry to breed pigs with better meat quality. Studies demonstrate that meat pH and meat color are closely connected with the quality of meat, affecting freshness, shearing force, and drip loss of pork [1,2,3]. Therefore, applying molecular genetic approaches to meat pH and color traits are useful strategies to improve the meat quality [4,5,6], and this can accelerate the breeding cycle in pigs. Meat pH and meat color (L*, lightness; a*, redness; b*, yellowness) of pork are low-heritability traits and varies among different populations [7,8], implying that meat quality is difficult to improve by traditional selection [9]. Due to high phenotyping costs of meat quality traits (because these phenotypes usually are measured post-mortem) and genotyping costs of large-scale pigs, genetic processes in improving pork meat quality were slow. The effort to identify quantitative trait loci (QTLs) and genetic markers has been ongoing for many years using genome-wide association study (GWAS), which made it possible to discern the genetic basis of meat pH and meat color traits. Genome-wide association studies are widely used to detect associations of genotypes with phenotypes by testing for differences in the allele frequency of single nucleotide polymorphisms (SNPs) between individuals [10]. To date, a large number of QTLs (>900) have been detected as being associated with pork meat pH and meat color, as reported in the pig QTL database [11]. Previous studies also report that the *PRKAG3* gene plays an important role in affecting pork meat pH and color [12,13,14]. Ma et al. [15] demonstrated that a causal loss-of-function mutation in the *PHKG1* gene causes high glycogen content and low meat quality in pigs. Although numerous QTLs and genes affecting meat pH and meat color traits have been identified [16,17,18], the genetic architecture of these polygenic quantitative traits remains elusive.

The aim of this study was to detect QTLs and genes associated with meat quality traits in pigs. To this end, we conducted GWAS for eight meat pH and meat color traits in a three-way crossbred commercial Duroc × (Landrace × Yorkshire) (DLY) pig population, numbering 1518 individuals. We detect numerous significant SNPs and promising candidate genes affecting meat quality and further reveal the complexity of the genetic architecture of meat quality. In terms of improving the genetic process of meat quality, the identified significant SNPs can be used for the genetic improvement of meat pH and color in pigs by assigning higher weights to associated SNPs in genomic selection. The expansion of the basis of meat quality can potentially provide novel insights into future molecular breeding of pork and achieve better meat quality.

## 2. Materials and Methods

### 2.1. Ethics Statement

All animals used in this study met the guidelines for the care and use of experimental animals established by the Ministry of Agriculture of China. Tissue samples from pigs were collected with the approval of the ethics committee of South China Agricultural University (Guangzhou, China) under 2018F098.

### 2.2. Animal Samples and Meat Quality Traits Phenotyping

The experiment animals were from a three-way crossbred commercial pig population. Briefly, 84 Duroc boars were mated to 397 Landrace × Yorkshire sows to produce a large-scale offspring. All pigs sustained uniform feeding conditions and were raised on four farms of Wen’s Foodstuffs Group Co., Ltd. (Guangdong, China). After fattening, 1518 individuals born from 2018 to 2019 were slaughtered for phenotype recording at an average body weight of 115 kg in 13 batches. For each animal, longissimus thoracis (LT) muscle was removed from the left side of each carcass and was used to determine meat quality traits. Meat pH and meat color traits measurements were performed on LT muscle as described previously [19]. Briefly, a portable pH meter (equipped with an insertion glass electrode) was used to determine the pH of LT muscle samples between the 11th and 12th ribs at 45 min (pH_45min) and 12h (pH_12h) after slaughter, respectively. The pH meter was calibrated before measurements using standard phosphate buffers (pH = 4.01 and 7.00) and adjusted to the actual temperature of sample measurement following the instrumental user’s manual. Three meat color parameters (L*, a*, and b*) were measured on the exposed cut surface after blooming for 30 min of the LT at 45 min and 12 h post-mortem, respectively, using a CM-2600d/2500d Minolta Chromameter (Tokyo, Japan) with an 8 mm measuring port, D65 illuminant, and one permanent observer to avoid human factor. Meat samples of LT were kept at 4 °C inside the refrigerator until the pH, L*, a*, and b* were measured at 12 h post-mortem.

### 2.3. Genotyping and Quality Control

Genomic DNA of each pig was extracted from ear tissue via a standard phenol/chloroform method and was diluted to 50 ng/μL [20] for genotyping procedure. The 1518 DLY pigs were genotyped using a GeneSeek Porcine 50K BeadChip (Neogen, Lincoln, NE, USA), which contained 50,703 SNPs. After genotyping, the genotype dataset was converted from Sus scrofa genome (*Sscrofa*) 10.2 to build *Sscrofa* 11.1. Quality control (QC) procedures were conducted using PLINK v1.07 software [21] with the following criteria: individual call rate > 95%, SNP call rate > 99%, minor allele frequency > 1%, and *p* > 10^−6^ for the Hardy–Weinberg equilibrium test. Only SNPs located on the autosome chromosomes were retained in this study. After QC, we removed 203 SNPs because of missing genotype data, 18,004 SNPs due to Hardy–Weinberg exact test, and 45 SNPs due to minor allele threshold. Moreover, 4245 SNPs not located on autosome chromosomes were discarded. Finally, 1518 pigs and 28,206 SNPs remained for subsequent analyses.

### 2.4. Population Structure and Linkage Disequilibrium Estimation

The qualified SNPs were used to conduct principal component analysis (PCA) and linkage disequilibrium (LD) analysis to investigate the population structure of the DLY pigs. PCA was conducted with GCTA software [22]. The SNPs distributed in the pig genomes were used to calculate the average LD decay distance, which was estimated as squared correlation of allele frequencies (*r*^2^). The window size of LD block was set as 1000 using PLINK v1.07 software.

### 2.5. Genome-Wide Association Study

We conducted GWAS with GEMMA software [23] for eight meat pH and meat color traits including pH_45min, L*_45min, a*_45min, b*_45min, pH_12h, L*_12h, a*_12h, and b*_12h. The mixed linear model used in this study was as follows:

(1) y=Wα+xβ+g+ε

where y is the vector of each meat quality trait in DLY pig population; W is a incidence matrix of covariates (fixed effects) including farms (four levels), sex (two levels), slaughter batches (13 levels), and the top five eigenvectors of PCs and a column of 1s; α is a vector of corresponding coefficients that includes the intercept; x is the vector of all SNP marker genotypes; β refers to the corresponding effect of the SNP; g refers to an n × 1 vector of random effects, with g ~ MVN (0, K$\sigma_{g}^{2}$), and ε is the vector of random residuals, with ε ~ MVN (0, I$\sigma_{e}^{2}$); K is genomic relatedness matrix and $\sigma_{g}^{2}$ is the additive genetic variance; I is the identity matrix and $\sigma_{e}^{2}$ is the residual variance; n refers to the number of analyzed DLY pigs; and MVN denotes multivariate normal distribution.

In the current study, we used Bonferroni correction to acquire the genome-wide significant and chromosome-wide significant thresholds. A SNP was considered to have genome-wide significance at *p* < 0.05/N and chromosome-wide significance at *p* < 1/N, where N refers to the total number of qualified SNPs. The quantile–quantile (Q–Q) plots of the eight meat quality traits were constructed for the assessment of population stratification effects on GWAS results.

We used Haploview v4.2 software [24] to perform haplotype block analysis within the identified significant QTLs.

### 2.6. Estimation of Heritability and Phenotypic Variance

In this study, we conducted SNP-based heritability estimation of each meat quality trait and calculated trait-associated SNP’s contribution to phenotypic variance via GCTA software [22]. The SNP-based heritability and the proportion of phenotypic variance explained by significant SNPs were calculated using:

(2) $$y=X\beta +g+\varepsilon \;\mathrm{with}\;\mathrm{var}\left(y\right)=A_{g}\sigma_{g}^{2}+Ι\sigma_{e}^{2}$$

where y is the phenotypic value of each meat quality trait; β is the vector including covariates as described above; X is an incidence matrix; g is an n × 1 vector of the total genetic effects of the analyzed pigs; Ι is the identity matrix; $A_{g}$ refers to the genomic relatedness matrix between animals; $\sigma_{g}^{2}$ is the additive genetic variance estimated by the restricted maximum likelihood approach; and $\sigma_{e}^{2}$ is the residual variance.

### 2.7. Functional Candidate Genes Search

The functional genes were searched for based on *Sscrofa* 11.1 genome version (http://asia.ensembl.org/Sus_scrofa/Info/Index, accessed on 9 July 2022). Genes nearest the significant SNPs are list in the Tables. Furthermore, we manually queried PubMed and the literature for information about the association between all candidate genes nearest peak SNPs and the analyzed meat quality traits.

## 3. Results and Discussion

### 3.1. Phenotype Statistic and Heritability Estimation

The descriptive statistics of eight meat quality traits in DLY pigs are listed in Table 1. For meat pH and meat color values, they are important parameters affecting sensory quality of pork. In this study, differences in phenotypic values are observed between the meat quality traits measured at 45 min and 12 h post-mortem. A similar pattern is also observed in three Chinese indigenous breeds [19]. Genetic factors that contribute to the phenotype variations in pH, L*, a*, and b* are explored (see below). The heritability of meat pH ranges from 0.12 to 0.17, while the heritability of meat color traits ranges from 0.03 to 0.12. As previous studies report [7,8], meat pH and meat color (L*, a*, b*) of pork are low-heritability traits. Miar et al. [8] report the heritability of meat color varies from 0.1 to 0.4, and pH is 0.15 in commercial crossbred pigs. Analyses performed in the present study indicate that the heritability of meat color traits in DLY pigs is lower than that reported in a previous study. For low-heritability traits in pigs, traditional breeding strategies have weak power in terms of improving the genetic process. Therefore, meat quality may be improved using genetic methods, such as genomic selection, to accelerate the breeding cycle in pigs [8].

### 3.2. Assessment of Population Structure and Linkage Disequilibrium Decay

**Figure 1:**
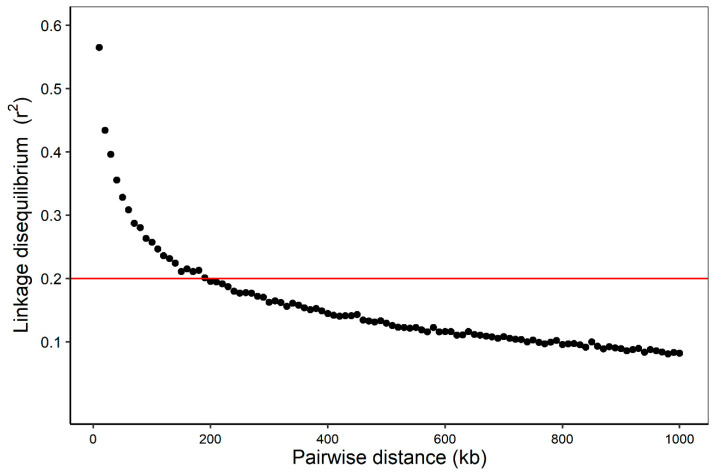
Linkage disequilibrium decay across the whole genome of the association panel. The red line represents the linkage disequilibrium threshold for the association panel (*r*^2^ = 0.2).

**Figure 2:**
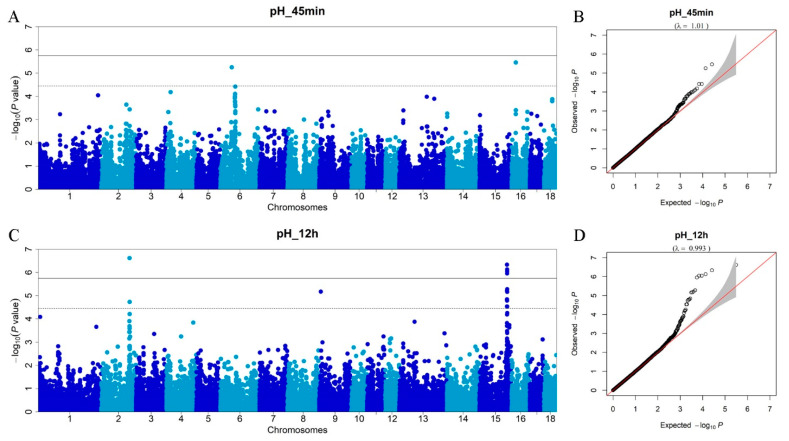
Manhattan plots of the GWAS and Q–Q plots for meat pH traits. (**A**) GWAS for pH_45min; (**B**) Q–Q plot for pH_45min; (**C**) GWAS for pH_12h; (**D**) Q–Q plot for pH_12h. The *x*-axis represents the chromosomes, and the *y*-axis represents the −log10 (*p*-value).

**Figure 3:**
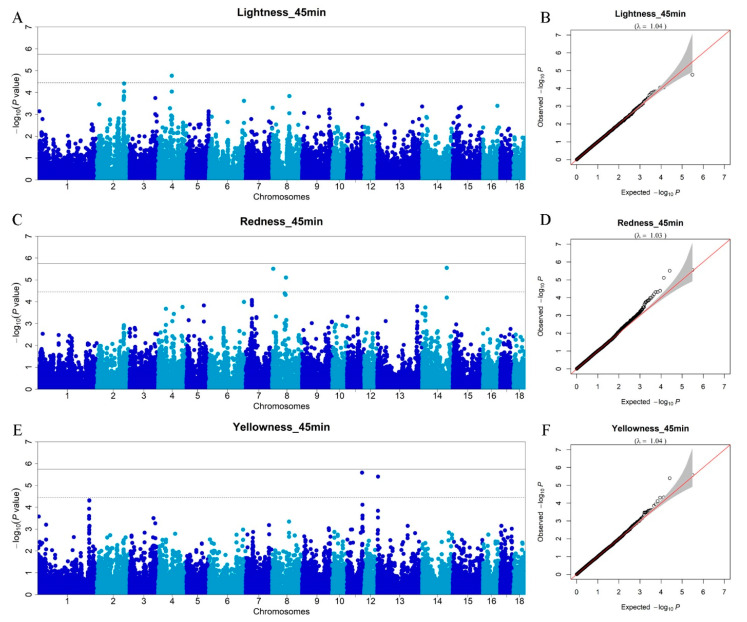
Manhattan plots of the GWAS and Q–Q plots for meat color traits (45 min). (**A**) GWAS for L*_45min (lightness_45min); (**B**) Q–Q plot for L*_45min (lightness_45min); (**C**) GWAS for a*_45min (redness_45min); (**D**) Q–Q plot for a*_45min (redness_45min); (**E**) GWAS for b*_45min (yellowness_45min); (**F**) Q–Q plot for b*_45min (yellowness_45min). The *x*-axis represents the chromosomes, and the *y*-axis represents the −log10 (*p*-value).

**Figure 4:**
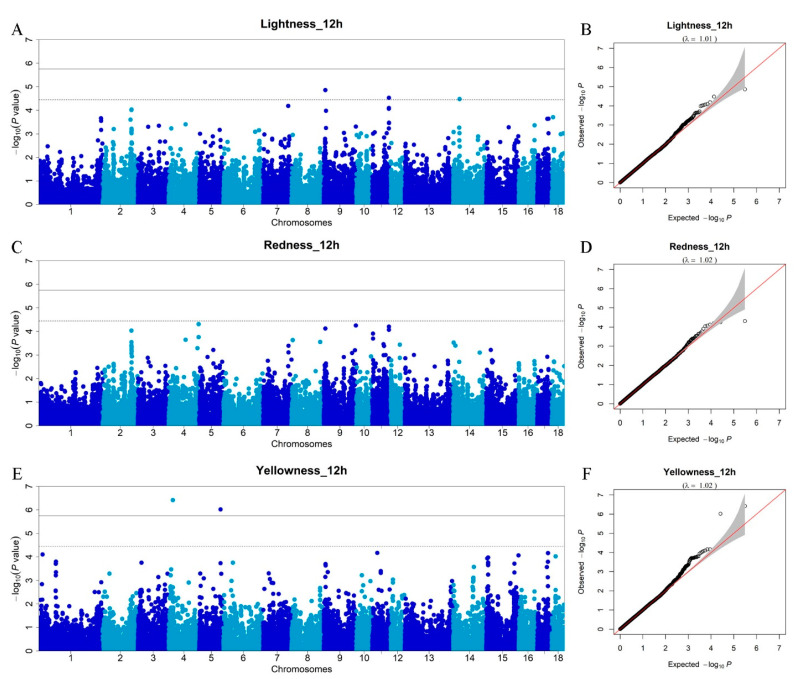
Manhattan plots of the GWAS and Q–Q plots for meat color traits (12 h). (**A**) GWAS for L*_12h (lightness_12h); (**B**) Q–Q plot for L*_12h (lightness_12h); (**C**) GWAS for a*_12h (redness_12h); (**D**) Q–Q plot for a*_12h (redness_12h; (**E**) GWAS for b*_12h (yellowness_12h); (**F**) Q–Q plot for b*_12h (yellowness_12h). The *x*-axis represents the chromosomes, and the *y*-axis represents the -log10 (*p*-value).

The average *r*^2^ of 0.2 is about 200 kb apart (Figure 1). Therefore, our three-way crossbreed DLY pig population has a low LD pattern [25,26], which is useful for mining QTLs affecting meat quality. In this study, we added the first five principal components into the association model as covariates to correct the potential population structure. As shown in the Q–Q plots (together with the Manhattan plots: Figure 2, Figure 3 and Figure 4), the genomic inflation factors (lambda) of the GWAS results for all traits are close to 1, which indicates no evidence of population stratification.

### 3.3. Genome-Wide Association Studies for Meat pH

The GWAS for pH_45min identifies two SNPs that are located on SSC6 and 16 (Figure 2A; Table 2). Figure 2B shows that the lambda is 1.01. These two SNPs surpass the chromosome-wide threshold (*p* < 3.56 × 10^−5^) and contribute to 2.36% and 1.27% of the phenotypic variance, respectively. The SNP (rs81274518) on SSC6 is located within the *GRIK5* gene and the SNP (rs81324442) on SSC16 is located within the *MTMR12* gene. *GRIK5* is a protein-coding gene that belongs to the glutamate-gated ionic channel family. This gene encodes glutamate ionotropic receptor kainite type subunit 5 in diverse ophthalmologic and vascular disorders when its expression levels are reduced [27]. *MTMR12* is a protein-coding gene and the protein encoded by this gene functions as an adaptor subunit in a complex with an active PtdIns(3)P 3-phosphatase. There is no study that highlights the *MTMR12* gene as a candidate gene related to meat quality traits in pigs. Significant SNPs detected by GWAS for meat pH_12h of the DLY pigs are shown in Table 3. In sum, 16 SNPs are found to be associated with pH_12h (Figure 2C). Figure 2D shows that the lambda is 0.993. Of these, 6 surpass the genome-wide threshold (*p* < 1.78 × 10^−6^) and 10 surpass the chromosome-wide threshold (*p* < 3.56 × 10^−5^). Notably, we detect one consistent QTL on SSC15 for meat quality, and the top SNP demonstrate the highest phenotypic variance of pH_12h in this QTL, at 2.67% (see below). We also identify three new SNPs associated with pH_12h, which are located on SSC2 and SSC9, respectively. The rs81303631 SNP and rs81295472 SNP on SSC2, which explain 0.72% and 0.31% of the phenotypic variance, respectively, are close to the protein-coding genes *FAM170A* and *PRR16*. The *FAM170A* gene acts as a nuclear transcription factor that positively regulates the expression of heat shock genes. The *PRR16* is a protein-coding gene involving in the positive regulation of cell size and positive regulation of translation [28]. *PRR16* is highly expressed in human placental endothelial cells. Although it is shown to regulate cell size, *PRR16*’s role in meat pH is unclear. The rs81316230 SNP on SSC9, which explains 0.31% of the phenotypic variance, is located inside the *PHOX2A* gene, which encodes a protein that contains a paired-like homeodomain most similar to that of the Drosophila aristaless gene product. It is shown that the *PHOX2A* gene is associated with respiratory rhythm and autonomic nervous system development [29].

### 3.4. Genome-Wide Association Studies for Meat Color Traits

In this study, we measured meat color parameters at two different time points (45 min and 12 h) after slaughter, which represent the meat color of fresh meat and chilled meat with different economic values.

In total, six SNPs (Table 2) in five chromosomes (SSC4, 8, 11, 13, and 14) surpass the chromosome-wide threshold (*p* < 3.56 × 10^−5^) and are found to be associated with three meat color traits (L*_45min, a*_45min, and b*_45min). For L*_45min, the SNP rs80971313 on SSC4 (Figure 3A), which explains 0.90% of the phenotypic variance, is located within the *KCNB2* gene. Figure 3B shows that the lambda is 1.04. The *KCNB2* gene is an important regulator of neuron excitability in mammal brains. It is reported that the *KCNB2* gene is involved in the muscular growth pathway that plays an important role in calcium and potassium transport, and in meat tenderization through its involvement in the proteolytic system responsible for post-mortem tenderization and muscle contraction [30,31]. However, post-mortem tenderization has an effect on pH decline during early post-mortem, which can affect other proteins and enzymatic systems that control meat quality characteristics such as meat color [32]. Therefore, we proposed the *KCNB2* gene as a promising candidate affecting meat color such as L*_45min. For a*_45min, three SNPs are detected (Figure 3C) and the lambda is 1.03 (Figure 3D). The strongest GWAS signal occurs on SSC14, and the peak SNP (rs80944912) is located within the *CNNM2* gene, but only explains 0.01% of the phenotypic variance, which implies a minor effect. Two SNPs are found to be significantly associated with b*_45min (Figure 3E) and the lambda is 1.04 (Figure 3F). The rs342146775 SNP on SSC11 is located within the *NALCN* gene and rs343103580 SNP on SSC13 is located within the *RFTN1* gene. *NALCN* is a protein-coding gene that plays an important role in insulin release [33]. Its related pathways are transport of glucose and other sugars, metal ions, and amine compounds. The *RFTN1* gene is involved in the B-cell receptor signaling pathway, membrane raft assembly, and positive regulation of growth rate. This gene is important in the formation or maintenance of membrane lipid rafts [34].

We performed GWAS in DLY pigs to detect SNPs and genes affecting meat color traits (12 h), including L*_12h, a*_12h, and b*_12h. In sum, five SNPs (Table 3) surpass the chromosome-wide threshold (*p* < 3.56 × 10^−5^) and are found to be associated with L*_12h and b*_12h. Three SNPs are associated with L*_12h (Figure 4A) and the lambda is 1.01 (Figure 4B). The rs81316230 SNP on SSC9, which explains 0.40% of the phenotypic variance, is close to the *PHOX2A* gene. This common result implies that rs81316230 SNP, together with the nearest gene *PHOX2A*, may have an effect on both meat color and pH. For a*_12h, no GWAS signal occurs on the autosomes (Figure 4C) and the lambda is 1.02 (Figure 4D). However, the rs81303631 SNP does not surpassed the chromosome-wide threshold, but is also highlighted as a candidate genetic marker for a*_12h due to its effect for pH_12h, implying that this SNP may have pleiotropic effects on meat quality in pigs. As for b*_12h, we identify two significant SNPs (Figure 4E) that exceed the genome-wide threshold (*p* < 1.78 × 10^−6^) and the lambda is 1.02 (Figure 4F). The rs343786555 SNP is located at 17.67 Mb near the *SHAS2* gene on SSC4. The rs346116771 SNP is close to the protein-encoding gene *EPYC*. However, these two SNPs have minor effect for b*_12h, which only contributes less than 0.09% of the phenotypic variance. In addition, it is reported that the expression of the *SHAS2* gene is significantly higher in baicalin-treated than control oocytes in the study related to pig oocytes and subsequent embryo development following parthenogenetic activation [35]. The *EPYC* gene is a member of the small leucine-rich repeat proteoglycan family. It regulates fibrillogenesis by interacting with collagen fibrils and other extracellular matrix proteins [36]. The potential role of the two genes in meat quality needs further investigation.

In this study, there are no overlapped GWAS signals among meat color traits measured in 45 min and 12 h. Our results demonstrate that the genetic factors affecting meat color in the two stages may be different. Meat color is an important economic trait and the genes causing the different meat color are shown in different pig breeds [37], implying that meat color is regulated by complex genetic networks.

### 3.5. Constant QTL for Meat pH on SSC15 Detected by GWAS

**Figure 5:**
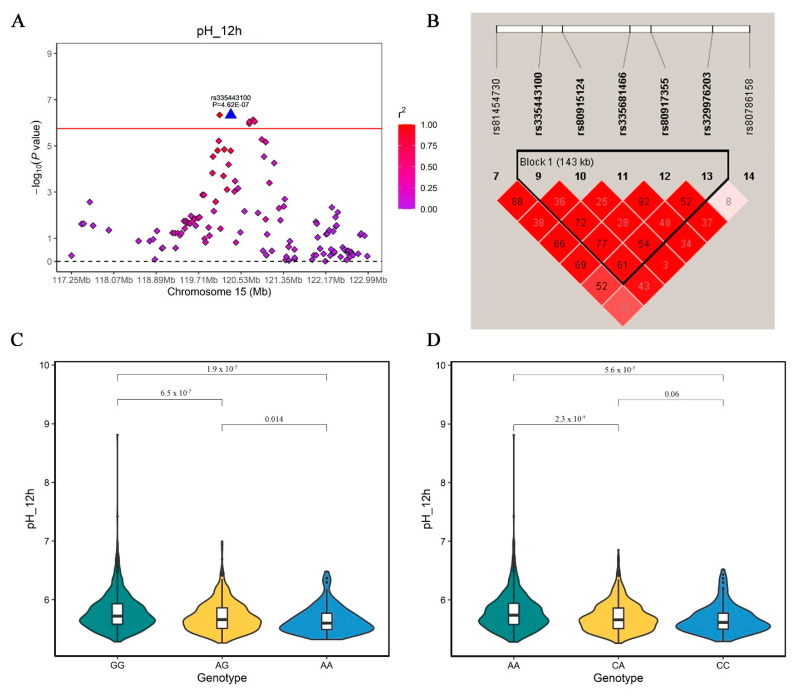
(**A**) Regional plots of rs335443100 at 117.25–122.99 Mb on SSC15 for meat pH_12h in DLY pigs. (**B**) represents the 143 kb linkage disequilibrium block in the significant region on SSC15. (**C**) Violin plot showing the differences in meat pH among three genotypes of the GWAS top SNP (rs335443100). (**D**) Violin plot showing the differences in meat pH among three genotypes of the SNP explaining the most phenotypic variance within *TTLL4* gene.

In this study, 13 SNPs associated with pH_12h are located in a QTL region on SSC15 between 117.25–122.99 Mb (*Sscofa* 11.1). Figure 5A is a region plot of this QTL and shows the LD pattern between the GWAS peak (rs335443100) and other significant SNPs. Notably, we detect one significant QTL on SSC15 with a 143 kb interval (Figure 5B) for pH_12h trait, together with the most promising candidate gene *TNS1*. The GWAS peak is approximately 733 kb away from the *PRKAG3* gene (also located on SSC15), a gene affecting glycogen in Hampshire and meat quality traits in pigs [12,13,14]. Based on the GWAS results in this study, the region around the *PRKAG3* gene gives a small *p*-value. Moreover, low LD (*r*^2^) is observed between the SNPs in the *TNS1* gene and *PRKAG3* region (Figure 5A). Beyond the *PRKAG3* gene, functional variants may exist in the identified QTL regions in the current study [38]. The top SNP (rs335443100) explains 1.94% phenotypic variance of the pH_12h trait. Previous studies report the potential influence of the *TNS1* gene on meat quality [39]. For instance, the *TNS1* gene is identified as a candidate in GWAS for pork meat pH measured 24 h after slaughter in Finnish Yorkshire pigs [38] and in Chinese Laiwu pigs [17]. The *TNS1* gene plays an important role in TGF-beta-induced myofibroblast differentiation [40], which indicates the involvement of this gene in the muscle development of pigs in the early period. These results imply that the *TNS1* gene on SSC15 of this QTL is likely to be responsible for meat pH. However, two SNPs located in the *TTLL4* gene explain the highest level of phenotypic variance of pH_12h in this QTL region, at 2.67%. We then evaluated the phenotype distribution pattern of the peak GWAS signal (Figure 5C) and the SNPs within the *TTLL4* gene (Figure 5D) in the DLY pigs. For the two SNPs, they have similar a decrease effect between GG vs. AA (GWAS peak: 5.79 vs. 5.65, respectively) and AA vs. CC (*TTLL4*: 5.79 vs. 5.67, respectively) genotype. However, the LD between the two SNPs is 0.48, implying an independent effect on meat pH by the *TTLL4* genes. Therefore, the *TTLL4* gene is proposed as another candidate gene responsible for meat pH in this QTL. *TTLL4* is a protein-coding gene and Gene Ontology annotations related to this gene include ligase activity and tubulin binding. It is involved in *KLF4* glutamylation, which impedes its ubiquitination, thereby leading to somatic cell reprogramming, pluripotency maintenance, and embryogenesis [41]. The potential role of the *TTLL4* gene in meat pH needs further investigation.

## 4. Conclusions

This study conducted a GWAS for eight meat quality traits in a 1518 DLY pig population, and provided valuable insights into elucidating the genetic architecture of meat pH and meat color. We identify one significant QTL on SSC15 with a 143 kb interval affecting pork pH, and two related genes (*TNS1*, *TTLL4*) are likely to be responsible for the meat pH difference among individuals according to their functions. From this, we detect several genetic markers affecting meat pH and color. Results from this study are useful for the genetic improvement of pork meat quality in swine by assigning higher weights to associated SNPs in genomic selection.

## Figures and Tables

**Table 1:** Summary statistics of meat quality traits in DLY pigs.

Traits	N	Mean (±SD)	*h*^2^ (±SE)
pH_45min	1480	6.33 ± 0.32	0.17 ± 0.03
L*_45min	1518	43.86 ± 2.42	0.12 ± 0.04
a*_45min	1516	−0.71 ± 1.02	0.03 ± 0.03
b*_45min	1517	7.31 ± 1.22	0.05 ± 0.03
pH_12h	1498	5.73 ± 0.28	0.12 ± 0.04
L*_12h	1515	48.4 ± 4.05	0.12 ± 0.04
a*_12h	1515	−0.46 ± 1.10	0.06 ± 0.03
b*_12h	1512	7.83 ± 1.45	0.03 ± 0.03

**Table 2:** Significant SNPs associated with meat quality traits (45 min) in DLY pigs.

Traits	Chr	SNP ID	Position (bp)	MAF	*p*-Value	R^2^ (%) ^a^	Nearest Gene	Distance (bp)
pH_45min	6	rs81274518	49883373	0.36	5.56 × 10^−6^	2.36	*GRIK5*	within
	16	rs81324442	18478368	0.46	3.46 × 10^−6^	1.27	*MTMR12*	within
L*_45min	4	rs80971313	63076056	0.40	1.70 × 10^−5^	0.90	*KCNB2*	within
a*_45min	8	rs332726079	5403889	0.22	3.09 × 10^−6^	0.10	*STK32B*	14440
	8	rs81400902	64388531	0.28	7.81 × 10^−6^	0.47	*/*	/
	14	rs80944912	113938495	0.11	2.81 × 10^−6^	0.01	*CNNM2*	within
b*_45min	11	rs342146775	69898991	0.42	2.59 × 10^−6^	1.01	*NALCN*	within
	13	rs343103580	3546365	0.12	3.95 × 10^−6^	0.37	*RFTN1*	within

^a^ Proportion of total phenotypic variation explained by each SNP.

**Table 3:** Significant SNPs associated with meat quality traits (12 h) in DLY pigs.

Traits	Chr	SNP ID	Position (bp)	MAF	*p*-Value	R^2^ (%)^a^	Nearest Gene	Distance (bp)
pH_12h	2	rs81303631	123600292	0.26	2.40 × 10^−7^	0.72	*FAM170A*	133583
	2	rs81295472	123998374	0.12	1.86 × 10^−5^	0.31	*PRR16*	201134
	9	rs81316230	6815282	0.23	6.68 × 10^−6^	0.31	*PHOX2A*	2903
	15	rs81454672	119995203	0.30	2.91 × 10^−5^	1.33	*TNS1*	within
	15	rs81454730	120083397	0.29	1.59 × 10^−5^	1.61	*TNS1*	within
	15	rs80818610	120106066	0.48	6.13 × 10^−6^	1.68	*TNS1*	within
	15	rs335443100	120121891	0.30	4.62 × 10^−7^	1.94	*TNS1*	within
	15	rs80917355	120213666	0.35	1.43 × 10^−5^	1.52	*RUFY4*	204
	15	rs338238642	120337815	0.33	1.63 × 10^−5^	1.61	*GPBAR1*	within
	15	rs80816788	120696351	0.33	1.10 × 10^−6^	2.52	*/*	/
	15	rs320130359	120699144	0.33	8.96 × 10^−7^	2.52	*/*	/
	15	/	120770590	0.32	7.44 × 10^−7^	2.67	*TTLL4*	2595
	15	/	120801238	0.32	8.91 × 10^−7^	2.67	*TTLL4*	within
	15	rs345318543	120938602	0.35	5.27 × 10^−6^	2.04	*WNT10A*	172
	15	/	120982452	0.32	2.93 × 10^−5^	1.89	*CDK5R2*	2793
	15	rs81218648	121014341	0.28	6.87 × 10^−6^	2.46	*CRYBA2*	within
L*_12h	9	rs81316230	6815282	0.23	1.38 × 10^−5^	0.40	*PHOX2A*	2903
	11	rs80993821	71619677	0.11	2.95 × 10^−5^	0.62	*SLC10A2*	270898
	14	rs80985792	27342214	0.20	3.32 × 10^−5^	0.98	*/*	/
a*_12h	2	rs81303631*	123600292	0.26	9.18 × 10^−5^	0.11	*FAM170A*	133583
b*_12h	4	rs343786555	17678626	0.08	3.84 × 10^−7^	0.01	*SHAS2*	3040
	5	rs346116771	92003197	0.05	9.56 × 10^−7^	0.09	*EPYC*	125750

^a^ Proportion of total phenotypic variation explained by each SNP.

## Data Availability

Data is contained within the article.
